# Large Language Models for Intraoperative Decision Support in Plastic Surgery: A Comparison between ChatGPT-4 and Gemini

**DOI:** 10.3390/medicina60060957

**Published:** 2024-06-08

**Authors:** Cesar A. Gomez-Cabello, Sahar Borna, Sophia M. Pressman, Syed Ali Haider, Antonio J. Forte

**Affiliations:** 1 Division of Plastic Surgery, Mayo Clinic, 4500 San Pablo Rd S, Jacksonville, FL 32224, USA; 2 Center for Digital Health, Mayo Clinic, 200 First St. SW, Rochester, MN 55905, USA

**Keywords:** large language models, ChatGPT, Gemini, artificial intelligence, intraoperative, plastic surgery, reconstructive surgery

## Abstract

*Background and Objectives:* Large language models (LLMs) are emerging as valuable tools in plastic surgery, potentially reducing surgeons’ cognitive loads and improving patients’ outcomes. This study aimed to assess and compare the current state of the two most common and readily available LLMs, Open AI’s ChatGPT-4 and Google’s Gemini Pro (1.0 Pro), in providing intraoperative decision support in plastic and reconstructive surgery procedures. *Materials and Methods:* We presented each LLM with 32 independent intraoperative scenarios spanning 5 procedures. We utilized a 5-point and a 3-point Likert scale for medical accuracy and relevance, respectively. We determined the readability of the responses using the Flesch–Kincaid Grade Level (FKGL) and Flesch Reading Ease (FRE) score. Additionally, we measured the models’ response time. We compared the performance using the Mann–Whitney U test and Student’s t-test. *Results:* ChatGPT-4 significantly outperformed Gemini in providing accurate (3.59 ± 0.84 vs. 3.13 ± 0.83, *p*-value = 0.022) and relevant (2.28 ± 0.77 vs. 1.88 ± 0.83, *p*-value = 0.032) responses. Alternatively, Gemini provided more concise and readable responses, with an average FKGL (12.80 ± 1.56) significantly lower than ChatGPT-4′s (15.00 ± 1.89) (*p* < 0.0001). However, there was no difference in the FRE scores (*p* = 0.174). Moreover, Gemini’s average response time was significantly faster (8.15 ± 1.42 s) than ChatGPT’-4′s (13.70 ± 2.87 s) (*p* < 0.0001). *Conclusions:* Although ChatGPT-4 provided more accurate and relevant responses, both models demonstrated potential as intraoperative tools. Nevertheless, their performance inconsistency across the different procedures underscores the need for further training and optimization to ensure their reliability as intraoperative decision-support tools.

## 1. Introduction

The introduction of artificial intelligence (AI) into medicine has revolutionized medical practice and patient management by offering precise and individualized healthcare delivery. The integration of deep-learning (DL) techniques into natural language processing (NLP) and the availability of vast amounts of public datasets has led to the development of large language models (LLMs) [1]. Using transformer architectures, LLMs can recognize, summarize, translate, predict, and generate text-based content from the knowledge gained from these extensive datasets [2]. With the increasing amount of medical data and the complexity of clinical decision-making, LLMs can be pivotal for improving the overall quality and efficiency of healthcare as they can assist physicians in making timely, informed decisions [3].

As in any other surgical specialty, plastic surgeons must make time-sensitive decisions that have a significant impact on a patient’s outcome and safety. They must maintain up-to-date and robust medical knowledge as well as solid cognitive and mechanical skills [4]. However, in one study of surgical errors, cognitive errors contributed to over half of the adverse events recorded, especially for less-experienced and sleep-deprived surgeons [4,5,6]. AI models can quickly process large quantities of data and demonstrate superior prediction and classification for decision-making [4,7], an advantageous ability intraoperatively. In their scoping review, Navarrete and Hashimoto [5] identified that the three most common uses of AI for intraoperative decision support were: (1) increasing the information available to surgeons, including retrieving similar cases; (2) accelerating intraoperative pathology, including tumor margin mapping, tumor classification, and tissue identification; and (3) recommending surgical steps. In theory, the former and the latter can easily be performed with the current LLMs.

LLMs can process audiovisual and multimodal data and learn their semantic relationships, enhancing machines’ capabilities to understand and generate human-like language [1,8,9]. In a study assessing ChatGPT’s medical accuracy and comprehensiveness, the model got >57% of the questions at least nearly all correct and 79% at least adequate [3]. In surgery, Oh et al. [10] showed that ChatGPT-4 attained a score of 76.4% in the Korean General Surgery Board Exam, with pediatric and breast knowledge scoring >80%. LLMs can help surgeons make more accurate decisions during surgery by providing alternative solutions to non-typical scenarios based on similar case studies or reference materials. For less-experienced surgeons, LLMs can be helpful with anatomical identification or variations, as well as next-surgical-step guidance [11,12,13]. By training LLMs on more extensive clinical data and medical literature, we might be able to develop AI systems that can support surgeons with intraoperative queries and difficulties, thereby reducing the cognitive load in the OR and contributing to improved patient safety [9,14].

Plastic surgery is a field of innovation where new techniques and research findings regularly emerge to improve patient-centered outcomes [14,15,16]. LLMs have been used in aesthetic surgery, general plastic surgery, craniofacial, microsurgery, and hand surgery, where they have displayed adequate knowledge for research proposals, patient counseling, clinical decision-making, and intraoperative support [2,16,17,18,19,20,21,22,23,24,25,26,27]. To evaluate ChatGPT’s knowledge of plastic surgery, Humar et al. [28] prompted the questions from the 2022 In-Service Examination, where the model surprisingly ranked in the 49th percentile for 1st-year integrated plastic surgery residents. However, it scored in the 13th percentile for 2nd-year residents and in the 0 percentile for 5th- and 6th-year residents.

In this study, we aim to evaluate and compare the current state of the two most common and readily available LLMs, Open AI’s ChatGPT-4 and Google’s Gemini, in providing intraoperative decision support in plastic and reconstructive surgery procedures without utilizing a retrieval-augmented generation (RAG) approach. Atkinson et al. [14] previously evaluated ChatGPT-4 for intraoperative support for complications in the Deep Inferior Perforator flap. Building on their research, we evaluated the potential of ChatGPT-4 and Gemini as adjunctive tools intraoperatively by analyzing their generalizability in common procedures in the major fields of plastic surgery: cosmetic, pediatric, craniofacial, microsurgery, general plastic surgery, and hand surgery.

## 2. Materials and Methods

### 2.1. Study Design

**Figure 1:**
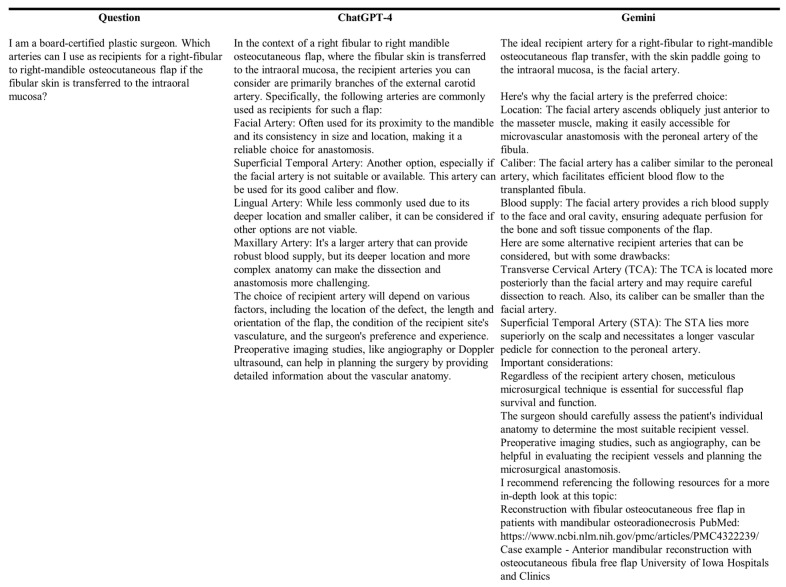
Example of the questions provided to the LLMs.

To evaluate the generalizability of the LLMs in plastic surgery, we created 32 scenarios ending in a question addressing surgical planning, general anatomy, surgical procedure knowledge, and the ability to provide solutions and alternatives for possible complications in 5 distinct procedures: breast augmentation (*n* = 6), complete cleft lip repair (*n* = 6), lymphaticovenous bypass (*n* = 8), mandibular reconstruction with fibula osteoseptocutaneous flap and osteomyocutaneous peroneal-artery-based combined flap harvest (*n* = 6), and carpal tunnel release (*n* = 6). Since the scenarios were not designed as if they were about a single patient per procedure, each was prompted individually in separate chats. All the questions were asked just once. After every scenario was presented to one model, the other was tested. Every scenario started with the statement “I am a board-certified plastic surgeon” and was narrated with medical terms to ensure that the models adequately narrowed their responses to the scenario. Additionally, the questions were brief in an attempt to simulate the time-sensitive setting of the OR. In Figure 1, we display an example of the scenarios presented to the LLMs, and in the Supplemental Files, we present the complete list.

### 2.2. Evaluation Tools

To evaluate the medical accuracy of the answers retrieved, we employed a 5-point Likert scale with the following values: 1 point: completely incorrect, the answer is entirely wrong and contradicts established medical knowledge; 2 points: partially incorrect, the answer has some validity but contains significant errors or misleading information; 3 points: partially correct and incorrect, there is a mix of correct and incorrect information; 4 points: partially correct, the answer contains some correct information but might be missing details or have minor inaccuracies; and 5 points: completely correct, the answer matches the information in reference textbooks and known practice. Alternatively, since the information could be medically accurate but still not pertinent or valuable for the intraoperative setting, we evaluated the relevance of the responses. We utilized a 3-point Likert scale where 1 point was irrelevant, meaning the answer did not provide useful information for the surgeon or the team; 2 points was somewhat relevant, meaning that the answer offered some general information but lacked specific guidance for the surgical situation; and 3 points was relevant, implying that the answer directly addressed the surgical scenario and provided helpful, actionable steps for the surgical team. For accuracy and relevance, we used as the ground truth surgical procedures from textbooks such as *Plastic Surgery: 6-Volume Set, 5th Edition*, *Grabb and Smith’s Plastic Surgery*, and *Green’s Hand Surgery* [29,30,31,32,33]. Three independent authors analyzed and graded the responses; the most common grade was utilized.

Because of the nature of the intraoperative setting, the responses provided should ideally be short and easy to read. We used the Flesch–Kincaid Grade Level (FKGL) and the Flesch Reading Ease (FRE) score to assess the readability and verbosity. The FKGL calculates a text’s approximate reading grade level, where a score of 8 indicates that the reader needs a grade 8 reading level or above to understand. The FRE provides a score between 1 and 100, with higher scores meaning that the document is easier to read. Both tests take into consideration the number of sentences, words, and syllables to produce a score, thus measuring verbosity [34].

Lastly, we measured the response time for each answer. While LLMs can provide almost instantaneous responses, we wanted to analyze the actual response time and compare it between the two models. Emphasizing the time-sensitive nature of the OR, we wanted to evaluate the consistency of the models in providing timely responses. We timed each response from when the prompt was sent to when the LLM finished providing the complete answer.

### 2.3. Statistical Analysis

We calculated and charted the mean, mode, standard deviation (SD), and range for all the evaluated metrics of the models’ responses using a Microsoft Excel spreadsheet ((Version 2403 Build 16.0.17425.20236) 64-bit). We used the Mann–Whitney U test to compare the models’ accuracy and relevance. For the readability and response time, we used a two-sample, unpaired, bilateral Student’s *t*-test. The Mann–Whitney U was calculated manually, while the Student’s t-test was calculated using Microsoft Excel’s statistical package. We considered a *p*-value < 0.05 to be statistically significant.

## 3. Results

### 3.1. Medical Accuracy

**Figure 2:**
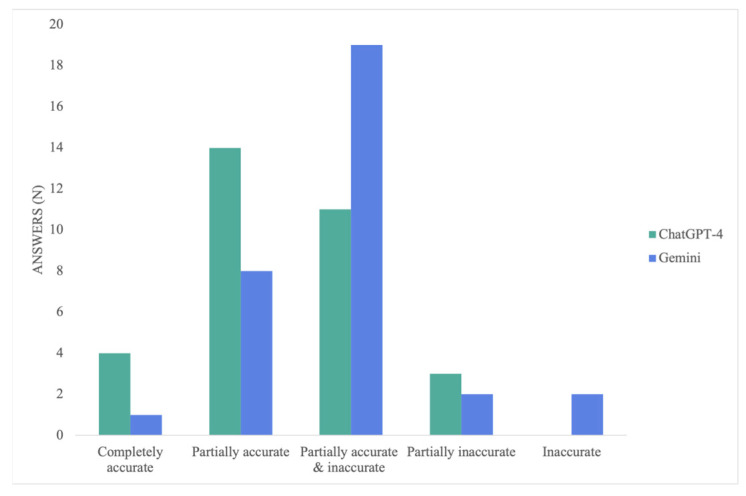
Accuracy scores per LLM.

Overall, ChatGPT-4’s responses were significantly more accurate than Gemini’s (*p* = 0.022). ChatGPT’s average mean score was 3.59 ± 0.84, with responses ranging from 2 to 5 points, and 56% of them were at least partially correct. Conversely, Gemini averaged 3.13 ± 0.83, with only 28% of its responses at least partially correct (≥4 points), 59% partially correct and incorrect (3 points), and scored as low as 1 point (completely incorrect) (Figure 2). ChatGPT-4 outperformed Gemini in all the procedures except for cheiloplasty, where 66% of Gemini’s and 50% of GPT’s responses scored three points; both got only one response partially correct and not even one entirely correct. However, the difference was not statistically significant (*p* = 0.344).

ChatGPT-4’s mean score of 4.00 ± 0.63 for breast augmentation was significantly better than Gemini’s 3 ± 1.00, with a *p*-value of 0.046. On average, 83% of ChatGPT-4’s responses were at least partially correct, while 50% of Gemini’s were partially correct and incorrect, 33% were partially accurate, and none were completely correct. For the lymphovenous bypass procedure, not only was there no significant difference (*p* = 0.200) but the performance was the most similar, with ChatGPT-4 averaging 3.75 ± 0.46 and Gemini 3.50 ± 0.53. Nevertheless, 75% of the former’s responses were partially correct. In comparison, only 50% of Gemini’s had the same result. No model provided either partially or incorrect answers or completely accurate responses.

**Figure 3:**
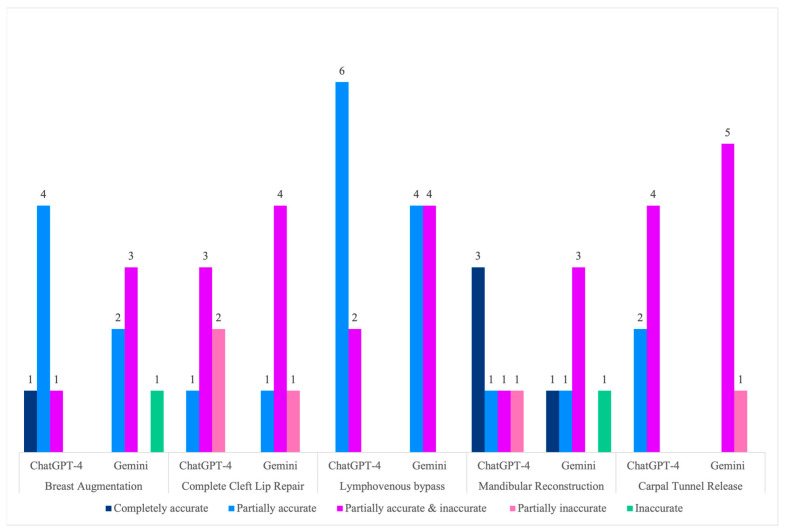
Accuracy scores per procedure.

There was no significant difference in the mandibular reconstruction with the fibular osteoseptocutaneous flap procedure (*p* = 0.149). However, 66% of ChatGPT-4’s responses were at least partially correct, with an average score of 4.00 ± 1.26, while 50% of Gemini’s were partially correct and incorrect, with a mean score of 3.17 ± 1.33. Notably, this was the only procedure where Gemini provided a completely correct answer. For carpal tunnel release, the average accuracy score for ChatGPT-4 was 3.33 ± 0.52, and for Gemini, 2.83 ± 0.41. A total of 83% of Gemini’s responses were partially correct and incorrect; meanwhile, 100% of ChatGPT-4’s were either partially correct and incorrect or partially correct. However, there was no statistically significant difference, with a *p*-value of 0.100. An overview of the models’ performance per procedure is shown in Figure 3.

### 3.2. Relevance

**Figure 4:**
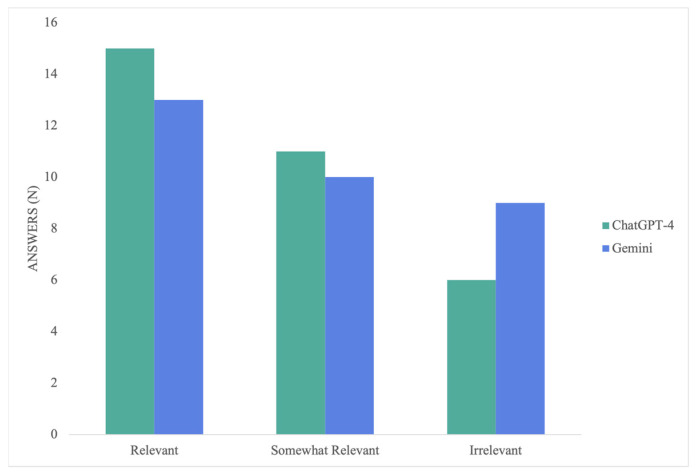
Relevance scores per LLM.

ChatGPT-4 significantly outperformed Gemini in terms of the answers’ relevance, with a *p*-value of 0.032. ChatGPT-4’s responses averaged 2.28 ± 0.77, ranging from irrelevant to relevant (1–3 points), with 47% being relevant. On the other hand, Gemini’s answers averaged 1.88 ± 0.83, and although they similarly ranged from 1 to 3 points, 40% were irrelevant (Figure 4). Similar to the accuracy, cheiloplasty was the only procedure where Gemini (1.67 ± 0.82) outperformed ChatGPT-4 (1.33 ± 1.00), providing 50% irrelevant responses and one relevant response. Conversely, ChatGPT-4 provided 66% irrelevant answers, and the rest were somewhat relevant. Nevertheless, there was no significant difference, with a *p*-value of 0.260.

While ChatGPT-4 was superior for the rest of the procedures, there was a significant difference for only carpal tunnel release (*p* = 0.015). The model achieved a mean score of 2.33 ± 0.82, and 83% of the responses were at least somewhat relevant. Gemini averaged 1.17 ± 0.41, and 83% of the responses were irrelevant. In terms of breast augmentation, ChatGPT-4’s mean score was 2.50 ± 0.55, 50% of its responses were relevant, and the other 50% were somewhat relevant. In contrast, Gemini averaged 1.83 ± 0.75 and provided somewhat relevant or irrelevant responses in 83% of the scenarios. However, there was no statistically significant difference (*p* = 0.075).

**Figure 5:**
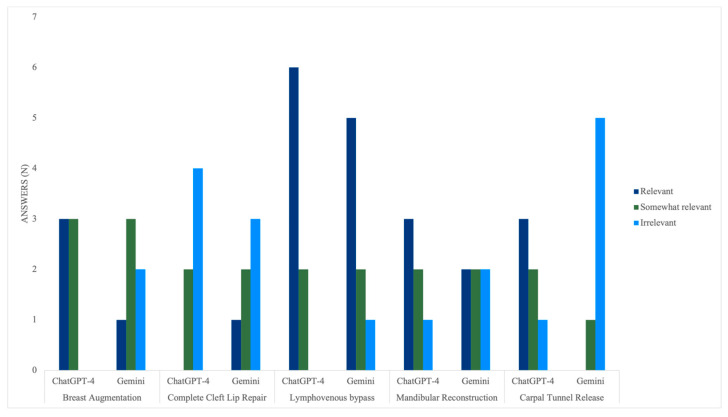
Relevance scores per procedure.

Once more, the models’ performance was the most similar in the lymphovenous bypass procedure, with mean scores of 2.75 ± 0.46 and 2.50 ± 0.76 for ChatGPT-4 and Gemini, respectively. There was no significant difference between the two (*p* = 0.299). Nonetheless, ChatGPT-4 proved its superiority by providing relevant responses in six out of eight scenarios and no irrelevant responses. Gemini, on the other side, provided five relevant responses and one irrelevant response. In the mandibular reconstruction with fibular osteoseptocutaneous flap procedure, ChatGPT-4 had an average score of 2.33 ± 0.82 and retrieved relevant responses 50% of the time. Conversely, Gemini averaged 2.00 ± 0.89 and provided relevant responses 33% of the time. There was no significant difference for this procedure (*p* = 0.260) (Figure 5).

### 3.3. Readability

Gemini’s responses were more readable and concise, most of the time also being shorter. This was shown by Gemini’s significantly lower FKGL mean of 12.80 ± 1.56 compared to ChatGPT-4’s mean of 15.00 ± 1.89, with a *p*-value < 0.0001. Gemini’s superiority in terms of readability was also statistically significant in three of the procedures: breast augmentation: 12.22 ± 1.47 (Gemini) vs. 14.47 ± 1.11 (ChatGPT-4), *p*-value 0.013; lymphaticovenous bypass: 12.40 ± 1.55 (Gemini) vs. 16.10 ± 1.71 (ChatGPT-4), *p*-value < 0.001; and mandibular reconstruction: 14.33 ± 1.19 (Gemini) vs. 16.67 ± 1.59 (ChatGPT-4), with a *p*-value of 0.016. For the two remaining procedures, while Gemini averaged lower scores, there was no significant difference. In the complete cleft lip repair, Gemini’s mean score was 11.88 ± 1.50, and ChatGPT-4’s was 13.32 ± 1.73 (*p* = 0.156). In the carpal tunnel release procedure, Gemini averaged 13.30 ± 1.10 while ChatGPT-4 averaged 14.07 ± 1.13 (*p* = 0.261).

However, while Gemini’s average FRE score (23.75 ± 8.24) was higher than ChatGPT -4’s (24.06 ± 10.73), there was no statistically significant difference (*p* = 0.174). The only procedure where the average FRE score was significantly higher was the lymphovenous bypass (*p* = 0.050), as Gemini averaged 26.20 ± 7.38 and ChatGPT-4 averaged 17.46 ± 8.90. In breast augmentation, Gemini averaged 33.00 ± 8.94 and ChatGPT-4 averaged 26.20 ± 2.89 (*p* = 0.126). In cheiloplasty, Gemini’s mean score was 33.87 ± 4.95, and ChatGPT-4’s was 35.55 ± 12.06 (*p* = 0.758). In mandibular reconstruction, Gemini scored 19.33 ± 6.75, while ChatGPT-4 scored 16.90 ± 6.48 (*p* = 0.538). Finally, in carpal tunnel release, ChatGPT-4 provided more readable responses than Gemini (26.38 ± 10.25 vs. 24.73 ± 4.23). However, this was not statistically significant (*p* = 0.723).

### 3.4. Time of Response

**Figure 6:**
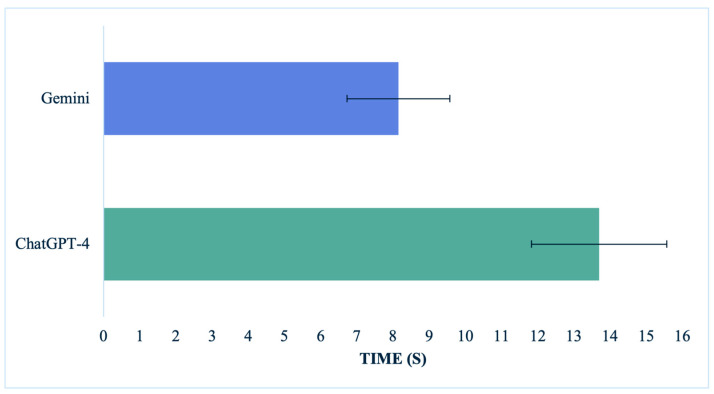
LLMs’ average response time in seconds. Error bars represent standard deviations.

Gemini significantly outperformed ChatGPT-4 in providing timely responses, with a *p*-value < 0.0001. The average response time for Gemini was 8.15 ± 1.42 s; meanwhile, ChatGPT-4’s average was 13.70 ± 2.87 s. For all the procedures, Gemini retrieved significantly faster responses than ChatGPT-4, with *p*-values < 0.001 for breast augmentation, lymphovenous bypass, and mandibular reconstruction and a *p*-value of 0.003 for cheiloplasty and 0.016 for carpal tunnel release. Gemini’s fastest performance was 4.78 s, provided in a mandibular reconstruction scenario, and its slowest was 11.13 s for a cleft lip repair question. Conversely, ChatGPT-4’s fastest response was provided for a carpal tunnel release scenario in 8.42 s, and its slowest response on a mandibular reconstruction case was 20.41 s. Figure 6 shows a comparison between the two LLMs. We present the complete list of the LLMs’ responses in the Supplementary File.

## 4. Discussion

In plastic surgery, LLMs are recognized due to their constant advancement and adequate medical performance [2,14,16,22,24,28,35]. Nevertheless, due to the inherent conditions of the intraoperative environment, in most scenarios, the margin of error is near 0. Moreover, these models’ use was previously limited as they were bound to text-only input and output, consuming valuable time during the surgical procedure. The new updates to ChatGPT and Gemini allow the models to receive and provide information with audio, which might improve the effectiveness of their use during the intraoperative period. Although the current experimentation of LLMs as intraoperative tools is limited, their application is promising.

Due to their ability to process vast amounts of data in several formats, LLMs can help with intraoperative monitoring and alert surgeons to intervene in a timely fashion [9,11,12,13]. They can also suggest individualized procedural modifications based on the latest research and clinical guidelines [14,20] while automatically generating surgical records with key information from the procedure [12,13]. LLMs may be groundbreaking for improving surgical outcomes by complementing human expertise and enhancing coordination with other AI instruments [9,12]. Ultimately, their responses can enhance the efficiency and precision of surgery as they connect theoretical knowledge and real-time surgical application [14].

This is the first study evaluating the current state of ChatGPT-4 and Gemini as intraoperative decision-support tools in plastic surgery without using any RAG technique. Furthermore, we compared the two models to identify their strengths and limitations and determine which was superior. For providing medically accurate information, ChatGPT-4 outperformed Gemini, whose answers were determined to be both partially correct and incorrect almost 60% of the time. While ChatGPT-4’s responses were more accurate, most of them were still determined to be only partially correct. Nevertheless, in most scenarios, both models proved an understanding of the situation about which they were questioned and retrieved concise and logical responses.

Similar to the results obtained by Atkinson et al. [14], the language models demonstrated an adequate understanding of anatomy most of the time, being able to help identify anatomical structures and landmarks to guide the surgeon during the procedure. This was especially noticeable during the lymphaticovenous bypass, mandibular reconstruction, and breast augmentation procedures. During the former, the models accurately recommended where to place the incisions when the ICG was not enough to find healthy lymphatics based on the understanding of the lymphatic and venous organization and their anatomical relationship. Moreover, the models were able to adequately suggest the appropriate location of the nourishing vessels for the fibula osteoseptocutaneous flap and provide useful recommendations for correctly identifying the major pectoral muscle for submuscular breast implant placement.

However, this performance was inconsistent, especially for ChatGPT-4. The model particularly struggled with the complete cleft lip repair, starting from the inability to accurately identify the anatomical locations for marking placement. Although ChatGPT-4 adequately recommended using the facial artery as a recipient for the fibula osteoseptocutaneous flap, it also recommended using the lingual and maxillary artery. Additionally, it also misunderstood the setting and provided irrelevant recommendations, where instead of offering actionable guidance, it recommended some literature. Gemini was somewhat better, notably for the cheiloplasty. It also provided more relevant responses. An example was seen in the mandibular reconstruction, where despite only recommending the facial artery as a recipient vessel, it provided a logical explanation for its reasoning, explaining why to discard other vessels, and recommended supplemental articles.

When it came to procedural steps, general procedure knowledge, and complication solving, ChatGPT-4 outperformed Gemini. Nonetheless, both models showed a strong grasp of the procedures and offered concise and logical guidance throughout. ChatGPT-4 particularly excelled in the breast augmentation and jaw reconstruction scenarios, while Gemini excelled in the complete cleft lip repair. They achieved the most similar performance during the lymphaticovenous bypass scenarios, where the models mostly provided both accurate and relevant responses. Conversely, their worst performance was on the carpal tunnel release procedure, where most of ChatGPT-4’s responses were superficial or incomplete, and those of Gemini’s were also irrelevant as they did not offer immediate solutions. This was consistent with another study evaluating ChatGPT for providing carpal tunnel syndrome diagnosis and management, where its responses were superficial, with no deeper explanation or reasoning for specific treatments, and referenced nonexistent publications [24].

Additionally, ChatGPT-4 kept struggling in terms of the cheiloplasty procedure, erroneously recommending pursuing a Mohler’s incision for a philtral column discrepancy of 1.7 mm without providing any reasoning. On the other hand, when asked about the recommended ischemia time limit for the fibular osteocutaneous flap, Gemini incorrectly advised that it was safe to exceed 5 h. Moreover, Gemini provided irrelevant responses to 40% of the questions, many of which were related to the model, stating that it was unable to provide medical advice and limiting its answers to recommending related literature or websites.

The models provided superficial and incomplete responses even when they were accurate, and while they were enough for some scenarios presented, for others they were not. In Mohapatra et al. [2], ChatGPT was evaluated as a teaching assistant for plastic surgery residents, and the authors concluded that the model was likely to cause confusion among residents as, despite providing fairly accurate procedural steps, it also provided inaccurate statements and missed critical steps. Additionally, Atkinson et al. [14] identified that although ChatGPT’s responses were consistently accurate, they were somewhat superficial and corresponded to the knowledge level of a trainee, not offering any insight beyond what an expert plastic surgeon would already know. 

Given the time-sensitive nature of the intraoperative scenarios, LLMs must provide concise and fast responses so that the surgical team can act timely. The FKGL and the FRE scores consider the number of sentences and words to determine a text’s reading level. In a previous study, ChatGPT’s FKGL and FRE score indicated a hard reading level appropriate for only 33% of adults and those with a college education [36]. Furthermore, the texts produced by ChatGPT were harder than those from Bard, Gemini’s predecessor [37]. Our results show similar characteristics, as Gemini’s FKGL was significantly lower than ChatGPT-4’s, and although there was no significant difference in the FRE score, Gemini’s was higher than ChatGPT-4’s, indicating that Gemini’s responses were easier to read. Even though a surgeon surpasses the level of education required for comprehending either model’s text, more readability also indicates more concise, straightforward responses. This came in conjunction with timelier responses, as Gemini proved to respond significantly faster than ChatGPT-4. However, Gemini’s evasiveness when responding may contribute to the difference in average readability scores and time of response.

The use of LLMs as intraoperative decision-support tools has significant ethical implications. Despite the models’ accurate and relevant performance, the lack of depth and consistency in their responses can lead to patient harm. This may raise issues of accountability and responsibility, as liability remains uncertain in the case of a negative outcome due to erroneous LLM guidance. Notably, Gemini states its inability to provide medical advice before continuing further with its response, while this is not the case with ChatGPT-4, which directly provides its answer. Moreover, there is the issue of data privacy and security, as current LLMs share all the information within their chats to their servers, but to be integrated into real clinical settings, they would need to handle sensitive patient information [38]. Additionally, as the models’ responses are based on their training data, they are subject to biases, posing the risk of outdated information, unequal care quality, and discrimination [39,40].

## 5. Strengths and Limitations

This is the first study comparing the current state of two of the most common and readily available LLMs as intraoperative decision-support tools in plastic and reconstructive surgery. By providing scenarios evaluating the models’ knowledge of anatomy, procedural steps, and problem-solving concerning five different procedures, we analyzed the models’ generalizability in the specialty. However, our study has some limitations. First is the limited number of questions per procedure, which limited the depth with which we could explore the model’s understanding of the procedures. Additionally, the design of the scenarios evaluating one procedure in independent patients limited our capacity to replicate and test the models’ ability to adapt to different complications in the dynamic and multifaceted nature of real-time surgical decision-making, where multiple factors and changing conditions must be considered simultaneously. Finally, the relatively small sample size may affect the power to detect significant differences between the LLMs, especially in the subgroup analyses of individual procedures. While our findings provide a strong foundation for understanding LLMs’ performance in plastic surgery, their generalizability to other surgical specialties needs further investigation. The current limitations are likely influenced by the models’ training data, which may not be equally comprehensive across other surgical domains. On the other hand, some limitations may be generalizable, such as the models’ struggle to understand the context due to flaws with the prompting. Using advanced prompting techniques could have avoided the superficiality and evasiveness of the models’ responses and helped improve their accuracy and relevance. By tailoring the prompts to the specific terminology and information needs of different surgical specialties, we can investigate whether these techniques can improve the overall accuracy, relevance, and applicability of LLMs for surgeons across a broader range of procedures.

It is important to consider that LLMs evolve rapidly and constantly, potentially limiting the truthfulness of our results in the near future. Nevertheless, the continuous evaluation of these models’ performance provides crucial insights to guide their future development and implementation in practice. Future research directions point toward the development of specialty-specific models leveraging fine-tuning techniques, such as retrieval-augmented generation, that allow restraining the models to good, accurate information and improve their contextual understanding.

## 6. Conclusions

Our study provided valuable insights into the current state of two readily available LLMs. Although ChatGPT-4 generally provided more medically accurate and relevant responses than Gemini, both models demonstrated adequate knowledge for supporting surgeons during operative procedures. However, the performance of both LLMs varied across the different surgical scenarios, with neither model consistently delivering completely correct or relevant information. This variability highlights the need for further development and optimization to ensure their reliability and precision in the intraoperative setting. This study underscored the critical balance between accuracy, relevance, speed, and conciseness that LLMs must achieve to be effectively integrated into this part of surgical practice. While the models have no immediate application, they still may provide valuable guidance, especially for inexperienced surgeons and residents. Additional experimentation leveraging retrieval-augmented generation techniques might help overcome the models’ current limitations and accelerate their application in the operating room.

## Data Availability

The raw data supporting the conclusions of this article will be made available by the authors upon request.

## Supplementary Materials

The following supporting information can be downloaded at https://www.mdpi.com/article/10.3390/medicina60060957/s1, Supplementary File S1: Complete Intraoperative Queries. Supplementary File S2: Complete set of LLM responses.

## References

[1] Hadi M.U., Al-Tashi Q., Qureshi R., Shah A., Muneer A., Irfan M., Zafar A., Shaikh M.B., Akhtar N., Al-Garadi M.A. (2023). Large Language Models: A Comprehensive Survey of Applications, Challenges, Limitations, and Future Prospects. Authorea Prepr..

[2] Mohapatra D.P., Thiruvoth F.M., Tripathy S., Rajan S., Vathulya M., Lakshmi P., Singh V.K., Haq A.U. (2023). Leveraging Large Language Models (LLM) for the Plastic Surgery Resident Training: Do They Have a Role?. Indian J. Plast. Surg..

[3] Johnson D., Goodman R., Patrinely J., Stone C., Zimmerman E., Donald R., Chang S., Berkowitz S., Finn A., Jahangir E. (2023). Assessing the Accuracy and Reliability of AI-Generated Medical Responses: An Evaluation of the Chat-GPT Model. Res Sq..

[4] Loftus T.J., Altieri M.S., Balch J.A., Abbott K.L., Choi J., Marwaha J.S., Hashimoto D.A., Brat G.A., Raftopoulos Y., Evans H.L. (2023). Artificial Intelligence-enabled Decision Support in Surgery: State-of-the-art and Future Directions. Ann. Surg..

[5] Navarrete-Welton A.J., Hashimoto D.A. (2020). Current applications of artificial intelligence for intraoperative decision support in surgery. Front. Med..

[6] Suliburk J.W., Buck Q.M., Pirko C.J., Massarweh N.N., Barshes N., Singh H., Rosengart T.K. (2019). Analysis of Human Performance Deficiencies Associated with Surgical Adverse Events. JAMA Netw. Open.

[7] Ren Y., Loftus T.J., Datta S., Ruppert M.M., Guan Z., Miao S., Shickel B., Feng Z., Giordano C., Upchurch G.R. (2022). Performance of a Machine Learning Algorithm Using Electronic Health Record Data to Predict Postoperative Complications and Report on a Mobile Platform. JAMA Netw. Open.

[8] Abi-Rafeh J., Henry N., Xu H.H., Bassiri-Tehrani B., Arezki A., Kazan R., Gilardino M.S., Nahai F. (2024). Utility and Comparative Performance of Current Artificial Intelligence Large Language Models as Postoperative Medical Support Chatbots in Aesthetic Surgery. Aesthet. Surg. J..

[9] He Y., Tang H., Wang D., Gu S., Ni G., Wu H. (2023). Will ChatGPT/GPT-4 be a Lighthouse to Guide Spinal Surgeons?. Ann. Biomed. Eng..

[10] Oh N., Choi G.S., Lee W.Y. (2023). ChatGPT goes to the operating room: Evaluating GPT-4 performance and its potential in surgical education and training in the era of large language models. Ann. Surg. Treat. Res..

[11] Cheng K., Li Z., Guo Q., Sun Z., Wu H., Li C. (2023). Emergency surgery in the era of artificial intelligence: ChatGPT could be the doctor’s right-hand man. Int. J. Surg..

[12] Cheng K., Sun Z., He Y., Gu S., Wu H. (2023). The potential impact of ChatGPT/GPT-4 on surgery: Will it topple the profession of surgeons?. Int. J. Surg..

[13] Li W., Zhang Y., Chen F. (2023). ChatGPT in Colorectal Surgery: A Promising Tool or a Passing Fad?. Ann. Biomed. Eng..

[14] Atkinson C.J., Seth I., Xie Y., Ross R.J., Hunter-Smith D.J., Rozen W.M., Cuomo R. (2024). Artificial Intelligence Language Model Performance for Rapid Intraoperative Queries in Plastic Surgery: ChatGPT and the Deep Inferior Epigastric Perforator Flap. J. Clin. Med..

[15] Gupta R., Pande P., Herzog I., Weisberger J., Chao J., Chaiyasate K., Lee E.S. (2023). Application of ChatGPT in Cosmetic Plastic Surgery: Ally or Antagonist?. Aesthet. Surg. J..

[16] Leypold T., Schäfer B., Boos A., Beier J. (2023). Can AI Think Like a Plastic Surgeon? Evaluating GPT-4’s Clinical Judgment in Reconstructive Procedures of the Upper Extremity. Plast. Reconstr. Surg. Glob. Open.

[17] Abi-Rafeh J., Hanna S., Bassiri-Tehrani B., Kazan R., Nahai F. (2023). Complications Following Facelift and Neck Lift: Implementation and Assessment of Large Language Model and Artificial Intelligence (ChatGPT) Performance Across 16 Simulated Patient Presentations. Aesthet. Plast. Surg..

[18] Abi-Rafeh J., Xu H.H., Kazan R., Tevlin R., Furnas H. (2024). Large Language Models and Artificial Intelligence: A Primer for Plastic Surgeons on the Demonstrated and Potential Applications, Promises, and Limitations of ChatGPT. Aesthet. Surg. J..

[19] Cox A., Seth I., Xie Y., Lang D., Hunter-Smith D.J., Rozen W.M. (2023). Utilizing ChatGPT-4 for Providing Medical Information on Blepharoplasties to Patients. Aesthet. Surg. J..

[20] Kwon D.Y., Wang A., Restrepo Mejia M., Saturno M.P., Oleru O., Seyidova N., Taub P.J. (2024). Adherence of a Large Language Model to Clinical Guidelines for Craniofacial Plastic and Reconstructive Surgeries. Ann. Plast. Surg..

[21] Liu H.Y., Alessandri-Bonetti M., Arellano J.A., Egro F.M. (2023). Can ChatGPT be the Plastic Surgeon’s New Digital Assistant? A Bibliometric Analysis and Scoping Review of ChatGPT in Plastic Surgery Literature. Aesthet. Plast. Surg..

[22] Seth I., Cox A., Xie Y., Bulloch G., Hunter-Smith D.J., Rozen W.M., Ross R.J. (2023). Evaluating Chatbot Efficacy for Answering Frequently Asked Questions in Plastic Surgery: A ChatGPT Case Study Focused on Breast Augmentation. Aesthet. Surg. J..

[23] Seth I., Lim B., Xie Y., Cevik J., Rozen W.M., Ross R.J., Lee M. (2023). Comparing the Efficacy of Large Language Models ChatGPT, BARD, and Bing AI in Providing Information on Rhinoplasty: An Observational Study. Aesthet. Surg. J. Open Forum.

[24] Seth I., Xie Y., Rodwell A., Gracias D., Bullock G., Hunter-Smith D.J., Rozen W.M. (2023). Exploring the Role of a Large Language Model on Carpal Tunnel Syndrome Management: An Observation Study of ChatGPT. J. Hand Surg. Am..

[25] Soto-Galindo G.A., Capelleras M., Cruellas M., Apaydin F. (2023). Effectiveness of ChatGPT in Identifying and Accurately Guiding Patients in Rhinoplasty Complications. Facial Plast. Surg..

[26] Vallurupalli M., Shah N.D., Vyas R.M. (2024). Validation of ChatGPT 3.5 as a Tool to Optimize Readability of Patient-facing Craniofacial Education Materials. Plast. Reconstr. Surg. Glob. Open.

[27] Yun J.Y., Kim D.J., Lee N., Kim E.K. (2023). A comprehensive evaluation of ChatGPT consultation quality for augmentation mammoplasty: A comparative analysis between plastic surgeons and laypersons. Int. J. Med. Inform..

[28] Humar P., Asaad M., Bengur F.B., Nguyen V. (2023). ChatGPT Is Equivalent to First-Year Plastic Surgery Residents: Evaluation of ChatGPT on the Plastic Surgery In-Service Examination. Aesthet. Surg. J..

[29] Wolfe S.W., Pederson W.C., Kozin S.H., Cohen M.S. (2022). Green’s Operative Hand Surgery 2-Volume Set.

[30] Loose J.E., Hopper R.A., Neligan P.C. (2024). Plastic Surgery: Volume 3: Craniofacial, Head and Neck Surgery and Pediatric Surgery.

[31] Song D.H., Hong J.P., Neligan P.C. (2024). Plastic Surgery: Volume 4: Lower Extremity, Trunk and Burns.

[32] Nahabedian M.Y., Neligan P.C. (2024). Plastic Surgery: Volume 5: Breast.

[33] Chung K. (2019). Grabb and Smith’s Plastic Surgery.

[34] Readable Flesch Reading Ease and the Flesch Kincaid Grade Level. 6 April 2024. https://readable.com/readability/flesch-reading-ease-flesch-kincaid-grade-level/.

[35] Copeland-Halperin L.R., O’Brien L., Copeland M. (2023). Evaluation of Artificial Intelligence-generated Responses to Common Plastic Surgery Questions. Plast. Reconstr. Surg. Glob. Open.

[36] Momenaei B., Wakabayashi T., Shahlaee A., Durrani A.F., Pandit S.A., Wang K., Mansour H.A., Abishek R.M., Xu D., Sridhar J. (2023). Appropriateness and Readability of ChatGPT-4-Generated Responses for Surgical Treatment of Retinal Diseases. Ophthalmol. Retina.

[37] Al-Sharif E.M., Penteado R.C., Dib El Jalbout N., Topilow N.J., Shoji M.K., Kikkawa D.O., Liu C.Y., Korn B.S. (2024). Evaluating the Accuracy of ChatGPT and Google BARD in Fielding Oculoplastic Patient Queries: A Comparative Study on Artificial versus Human Intelligence. Ophthalmic Plast. Reconstr. Surg..

[38] Yuan J., Tang R., Jiang X., Hu H. (2023). Large language models for healthcare data augmentation: An example on patient-trial matching. AMIA Annu. Symp. Proc..

[39] Leslie D., Mazumder A., Peppin A., Wolters M.K., Hagerty A. (2021). Does “AI” stand for augmenting inequality in the era of COVID-19 healthcare?. BMJ.

[40] Zaidi D., Miller T. (2023). Implicit Bias and Machine Learning in Health Care. South Med. J..

