# Supplementary material for: Large Language Models for Intraoperative Decision Support in Plastic Surgery: A Comparison between ChatGPT-4 and Gemini
Source: Medicina (Kaunas). 2024 Jun 8;60(6):957. doi: 10.3390/medicina60060957 (PMC11205293; doi:10.3390/medicina60060957)
Supplement: Supplementary file 1 [file medicina-60-00957-s001.zip › Supplemental file S1. Complete Intraoperative Queries.pdf]

Supplemental file 1. Complete Intraoperative Queries

Aesthetic—Breast Augmentation:

1. I am a board-certified plastic surgeon about to start a subpectoral breast augmentation procedure using a 400 g anatomical microtextured implant. How big should I make my incision?
2. I am a board-certified plastic surgeon currently in the middle of a subpectoral breast augmentation procedure. I finished the initial subcutaneous fat dissection and reached the musculature. What muscles may I encounter?
3. I am a board-certified plastic surgeon currently in the middle of a subpectoral breast augmentation procedure. I finished the initial subcutaneous fat dissection and reached the musculature. How can I be sure which is the major pectoral muscle?
4. I am a board-certified plastic surgeon currently dissecting the superolateral aspect of the breast implant pocket for a subpectoral breast implant. How can I know if I dissected too far?
5. I am a board-certified plastic surgeon. How far medially should I dissect for the creation of the subpectoral implant pocket for breast augmentation?
6. I am a board-certified plastic surgeon. I have already introduced the breast implant, but the lateral pocket appears tight. What should I do next?

Pediatric PLS—Complete Cleft Lip Repair (Cheiloplasty)

1. I am a board-certified plastic surgeon about to start a rotation advancement cheiloplasty for a left complete unilateral cleft lip. Tell me where I should make my markings .
2. I am a board-certified plastic surgeon marking my patient for a rotation advancement cheiloplasty for a left complete unilateral cleft lip. Where should I mark point 4 or the Christa Philtri's Inferior Left Lateral?
3. I am a board-certified plastic surgeon in the middle of a rotation advancement cheiloplasty. There is a discrepancy of 1.7 mm between the right and the left philtral columns. Should I proceed with the Mohler's incision?
4. I am a board-certified plastic surgeon repairing a left complete unilateral cleft lip. How far into the nasal mucosa should I extend the incision of the cleft side for an adequate advancement flap?
5. I am a board-certified plastic surgeon performing a left complete unilateral cleft lip repair with a rotation advancement technique. The alar base of the cleft side is depressed sagittally. What should I do to ensure adequate nasal mucosal repair?
6. I am a board-certified plastic surgeon currently reconstructing the orbicularis muscle of a left complete unilateral cleft lip. How should I place my sutures?

Microsurgery—Lymphaticovenular bypass

1. I am a board-certified plastic surgeon about to start a lymphaticovenular bypass but I can't find any lymphatics with the ICG. Where should I place my incision to identify suitable lymph vessels?
2. I am a board-certified plastic surgeon. How deep should I dissect to identify venous and lymphatic vessels for a lymphovenous bypass?
3. I am a board-certified plastic surgeon in the middle of a lymphaticovenular bypass procedure. How many incisions should I make, or how can I know when to stop making incisions?
4. I am a board-certified plastic surgeon. For an adequate lymphaticovenous bypass, what type of anastomosis should I use for a slightly ectatic lymph vessel but with patent flow?
5. I am a board-certified plastic surgeon in the middle of a lymphovenous bypass procedure. How can I confirm the patency of the anastomosis?
6. I am a board-certified plastic surgeon in the middle of a lymphovenous bypass procedure but my anastomosis doesn't show any washout of the venous lumen. What should I do next?
7. I am a board-certified plastic surgeon in the middle of a lymphovenous bypass procedure but my anastomosis has not shown any washout of the venous lumen. How can I assess the patency of the anastomosis at this point?
8. I am a board-certified plastic surgeon. How many lymphaticovenous anastomoses should I aim for to ensure successful lymphatic flow?

Flaps—Mandibular Reconstruction with Fibula Osteoseptocutaneous Flap and w/ Osteomyocutaneous Peroneal-Artery-Based Combined Flap Harvest

1. I am a board-certified plastic surgeon. Which arteries can I use as recipients for a right-fibular to right-mandible osteocutaneous flap if the fibular skin is transferred to the intraoral mucosa?
2. I am a board-certified plastic surgeon. I am having issues finding the donor vessels for my fibular osteoseptocutaneous flap. Where should I look?
3. I am a board-certified plastic surgeon harvesting an OPAC flap for a mandibular reconstruction. How much soleus muscle can I harvest without impairing the donor site mobility?
4. I am a board-certified plastic surgeon and I am having issues identifying the adequate nourishing vessels for the soleus muscle for an OPAC flap for a mandibular reconstruction. Where should I look?
5. I am a board-certified plastic surgeon harvesting an OPAC flap for a mandibular reconstruction. If I am doing multiple osteotomies, what is the minimal fibular segment length I can use without compromising blood supply?
6. I am a board-certified plastic surgeon, what is the ischemia time limit for a fibular osteocutaneous flap?

Hand—Carpal Tunnel Release

1. I am a board-certified plastic surgeon. I just finished the initial incision for an open carpal tunnel release but I need more exposure. How should I extend my incision?
2. I am a board-certified plastic surgeon. How can I ensure an adequate exposure of the distal part of the transverse carpal ligament for an adequate carpal tunnel release?
3. I am a board-certified plastic surgeon. How far distally should I incise the transverse carpal ligament to ensure an adequate median nerve release?
4. I am a board-certified plastic surgeon currently in the middle of a carpal tunnel release but I cannot identify the complete distal part of the transverse carpal ligament. My patient is obese; what do you recommend I do?
5. I am a board-certified plastic surgeon performing a carpal tunnel release but I am having difficulties releasing the antebrachial fascia. What do you recommend I do?
6. I am a board-certified plastic surgeon in the middle of a carpal tunnel release for a patient with severe thenar muscle wasting. I am having issues identifying the recurrent motor branch of the median nerve. What should I do?
